# Editorial: Update on the diagnosis and surgical treatment of gynecological tumors

**DOI:** 10.3389/fsurg.2025.1665026

**Published:** 2025-09-09

**Authors:** S. Cianci, V. A. Capozzi, S. Restaino

**Affiliations:** ^1^ Department of Human Pathology of Adult and Childhood “G. Barresi” Unit of Gynecology and Obstetrics, University of Messina, Messina, Italy; ^2^ Department of Obstetrics and Gynecology, University of Parma, Parma, Italy; ^3^ Department of Maternal and Child Health, Obstetrics and Gynecology Clinic, Ospedale Santa Maria Della Misericordia, Azienda Sanitaria Universitaria Friuli Centrale, Udine, Italy

**Keywords:** gynecological cancer, gynecologic oncology, gynecologic surgery, cancer diagnosis, gynecology, endoscopy

**Editorial on the Research Topic**
Update on the diagnosis and surgical treatment of gynecological tumors

Gynecological tumors, including malignancies of the ovaries, uterus, cervix, vulva, and vagina, account for a significant portion of cancer-related morbidity and mortality among women worldwide (1). Recent advances in diagnostic techniques and surgical interventions have significantly impacted clinical practice by contributing to earlier detection, better risk stratification, and more personalized therapeutic strategies.

Advances in imaging technology have improved the accuracy of preoperative tumor characterization. These tools facilitate precise staging of gynecologic malignancies (1, 2). Simultaneously, molecular diagnostics and next-generation sequencing have become integral to the management of ovarian and endometrial cancers. Identification of pathogenic variants such as BRCA1/2, TP53, and mismatch repair (MMR) gene deficiencies allows for more precise classification and targeted treatment planning (3–5).

Emerging diagnostic modalities, such as liquid biopsies, offer a non-invasive way to detect circulating tumor DNA (ctDNA) and other tumor-derived markers, providing a promising approach for monitoring disease recurrence and therapeutic response in real time (6, 7). Other promising technologies are available in the form of non-coding RNAs (ncRNAs) in gynecologic malignancies, particularly endometrial cancer (7, 8). While they are still undergoing validation for widespread clinical use, early studies show considerable potential in gynecologic oncology.

Surgical treatment remains the primary modality for most gynecologic cancers. Over the past decade, a paradigm shift toward minimally invasive surgery (MIS) has occurred, with laparoscopic and robotic-assisted techniques being increasingly preferred due to their favorable perioperative outcomes. Studies have demonstrated equivalent oncologic safety for selected early-stage endometrial and cervical cancers, along with reduced morbidity and shorter recovery times (6–10; Cianci et al.).

Robotic-assisted surgery, in particular, has gained prominence due to its enhanced visualization, dexterity, and ergonomics. While the upfront costs remain a limiting factor, this technology continues to expand, particularly in high-volume centers. For complex cases, such as radical hysterectomy or staging for high-grade endometrial carcinoma, robotics offers technical advantages that may translate into improved functional outcomes, especially when nerve-sparing procedures are employed (9, 11).

The management of gynecologic cancers is inherently multidisciplinary. Tumor boards that integrate surgical oncologists, medical oncologists, pathologists, radiologists, and reproductive specialists have become essential for guiding individualized treatment plans. Concurrently, artificial intelligence is beginning to influence diagnostic imaging and risk prediction models, although clinical integration is still in its early stages.

The diagnosis and surgical treatment of gynecological tumors continue to evolve, driven by technological innovation, molecular medicine, and a growing emphasis on patient-centered care.

It was a pleasure for us to serve as Guest Editors of the *Frontiers in Surgery* Research Topic entitled “*Update on the Diagnosis and Surgical Treatment of Gynecological Tumors*.” Here, we explored the latest innovations and state-of-the-art advancements in the diagnosis and surgical management of gynecological cancers. A series of articles authored by experts in the field of gynecologic oncology has been presented.

We believe that the topics addressed in this Research Topic will be of interest to a broad audience, ranging from academic researchers to clinicians working in gynecologic oncology.

Han et al., in “*A newly modified nerve-sparing radical hysterectomy technique with analysis of short-term oncologic, surgical, and functional outcomes*,” proposed a modified surgical approach for the treatment of cervical cancer.

The second article, by Huang et al., “*Early Recurrence After Surgery in FIGO 2023 Stage I–III Endometrial Cancer: Characteristics and Risk Factors*,” focused on the management of patients undergoing surgery for endometrial cancer.

In the third article, “*Robot-Assisted Müllerian Compartment Resection for Cervical Cancer*,” Li et al. presented compelling data on the use of robotic surgery for the treatment of cervical cancer.

The fourth article by Rei et al., “*Ultrasound in Endometrial Cancer: Evaluating the Impact of Pre-Surgical Staging*,” assessed the role of ultrasound in the pre-operative management of patients with endometrial cancer.

The fifth article, by Chen et al., entitled “*Comparison of the Value of the GI-RADS and ADNEX Models in the Diagnosis of Adnexal Tumours by Junior Physicians*,” examined the diagnostic accuracy of these models when used by less experienced clinicians.

In the narrative review “*Surgical Management of Anastomotic Leakage Related to Ovarian Cancer Surgery*,” Restaino et al. offered an overview of current strategies for managing this complex surgical complication.

The seventh article, by Su et al., “*Effect of Different Treatment Modalities on the Prognosis of Patients with Stage IIIC Cervical Cancer*,” investigated therapeutic options for patients with advanced-stage cervical cancer.

Finally, the eighth article by Wang et al., entitled “*Clinicopathological Characteristics and Fertility-Preserving Treatment of Atypical Polypoid Adenomyoma*,” presented new evidence regarding this rare uterine lesion and its conservative management.

We would like to express our sincere thanks to all the authors for their valuable contributions. These articles provide significant insights and enrich the scientific community's understanding of gynecological oncology.
